# Challenges and Opportunities for Implementing Integrated Mental Health Care: A District Level Situation Analysis from Five Low- and Middle-Income Countries

**DOI:** 10.1371/journal.pone.0088437

**Published:** 2014-02-18

**Authors:** Charlotte Hanlon, Nagendra P. Luitel, Tasneem Kathree, Vaibhav Murhar, Sanjay Shrivasta, Girmay Medhin, Joshua Ssebunnya, Abebaw Fekadu, Rahul Shidhaye, Inge Petersen, Mark Jordans, Fred Kigozi, Graham Thornicroft, Vikram Patel, Mark Tomlinson, Crick Lund, Erica Breuer, Mary De Silva, Martin Prince

**Affiliations:** 1 Department of Psychiatry, School of Medicine, College of Health Sciences, Addis Ababa University, Addis Ababa, Ethiopia; 2 Transcultural Psychosocial Organization Nepal, Kathmandu, Nepal; 3 School of Psychology, University of KwaZulu-Natal, Durban, South Africa; 4 PRIME India team, Sangath Non-Governmental Organisation, Goa, India; 5 Aklilu-Lemma Institute of Pathobiology, Addis Ababa University, Addis Ababa, Ethiopia; 6 Butabika National Mental Hospital, Kampala, Uganda; 7 Centre for Mental Health, Public Health Foundation of India, New Delhi, India; 8 Department of Research and Development, HealthNet Transcultural Psychosocial Organisation, Amsterdam, The Netherlands; 9 Centre for Global Mental Health, London School of Hygiene and Tropical Medicine, London, United Kingdom; 10 Health Service and Population Research Department, Institute of Psychiatry, King's College London, London, United Kingdom; 11 Sangath Non-Governmental Organisation, Goa, India; 12 Centre for Public Mental Health, Department of Psychology, Stellenbosch University and Department of Psychiatry and Mental Health, University of Cape Town, Cape Town, South Africa; 13 Alan J Flisher Centre for Public Mental Health, Department of Psychiatry and Mental Health, University of Cape Town, Cape Town, South Africa; 14 Centre for Global Mental Health, London School of Hygiene and Tropical Medicine, London, United Kingdom; 15 Centre for Global Mental Health, Institute of Psychiatry, King's College London, London, United Kingdom; Iranian Institute for Health Sciences Research, ACECR, Iran (Islamic republic of)

## Abstract

**Background:**

Little is known about how to tailor implementation of mental health services in low- and middle-income countries (LMICs) to the diverse settings encountered within and between countries. In this paper we compare the baseline context, challenges and opportunities in districts in five LMICs (Ethiopia, India, Nepal, South Africa and Uganda) participating in the PRogramme for Improving Mental health carE (PRIME). The purpose was to inform development and implementation of a comprehensive district plan to integrate mental health into primary care.

**Methods:**

A situation analysis tool was developed for the study, drawing on existing tools and expert consensus. Cross-sectional information obtained was largely in the public domain in all five districts.

**Results:**

The PRIME study districts face substantial contextual and health system challenges many of which are common across sites. Reliable information on existing treatment coverage for mental disorders was unavailable. Particularly in the low-income countries, many health service organisational requirements for mental health care were absent, including specialist mental health professionals to support the service and reliable supplies of medication. Across all sites, community mental health literacy was low and there were no models of multi-sectoral working or collaborations with traditional or religious healers. Nonetheless health system opportunities were apparent. In each district there was potential to apply existing models of care for tuberculosis and HIV or non-communicable disorders, which have established mechanisms for detection of drop-out from care, outreach and adherence support. The extensive networks of community-based health workers and volunteers in most districts provide further opportunities to expand mental health care.

**Conclusions:**

The low level of baseline health system preparedness across sites underlines that interventions at the levels of health care organisation, health facility and community will all be essential for sustainable delivery of quality mental health care integrated into primary care.

## Introduction

The unmet need for mental health care is high in most low- and middle-income countries (LMICs) [1], [2]. In some LMICs, such as Ethiopia, only 10% of people with severe forms of mental disorders ever receive effective care [3]. The consequences of untreated mental disorders include suffering, diminished quality of life and disability [4], human rights abuses, stigma and discrimination [5], poverty [6], poor physical health and premature mortality [7]. In response to this gross neglect of people with mental disorders, the World Health Organization (WHO) launched the mental health Gap Action Programme (mhGAP) which advocates scaling up of mental health care through integration into primary health care (PHC) and general medical services [8]. Evidence-based packages of care for prioritised mental, neurological and substance use (MNS) disorders in non-specialised health care settings (the mhGAP-Intervention Guide; mhGAP-IG) have been developed to support this process [9]–[11].

**Figure 1:**
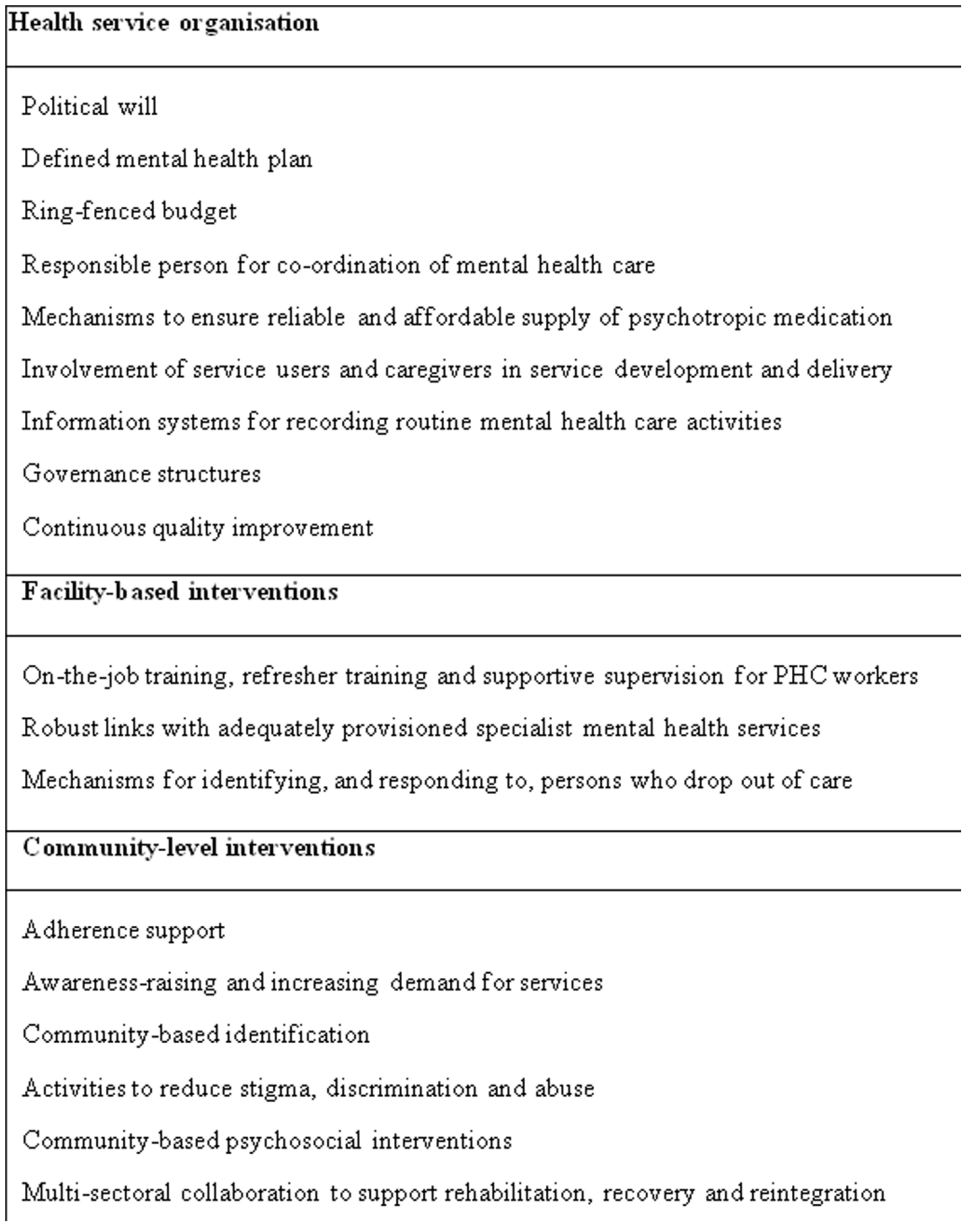
Requirements for integrating mental health into primary health care [14]–[17].

The decades of experience with attempts to integrate mental health care into PHC in LMICs have highlighted the challenges faced on the ground, particularly with respect to sustainability [12]. In order to maximise the beneficial impact of mhGAP, due attention needs to be paid to the process of ‘how to’ successfully implement and scale-up of mental health care in PHC [13]. Evidence and experience to date indicate that stand-alone training of PHC workers in mental health care is necessary but by no means sufficient to guarantee delivery [14]. Other components thought to be necessary are summarised in Figure 1 [14]–[17].

The Programme for Improvement of Mental health carE (PRIME) is operating in five LMICs (Ethiopia, India, Nepal, South Africa and Uganda) to provide evidence to support the implementation and scale-up of mental health care in primary care and maternal health care settings [18]. In each PRIME country a comprehensive mental health care plan will be developed for ‘districts’ (a geographically defined administrative unit for health service delivery), then implemented, evaluated and scaled-up. The PRIME mental health care plans will be multi-faceted and targeted at the three levels described above: health service organisation, health facility and community. The health facility level intervention will be based on the mhGAP-IG packages, adapted for the country context and restricted to priority MNS disorders selected for each country: psychosis, depression, alcohol use and maternal mental disorders in all PRIME countries, with epilepsy included additionally in Ethiopia, Nepal and Uganda.

We conducted a systematic baseline situation analysis in each of the five PRIME study sites, to describe the country-specific and cross-country factors relevant to development and implementation of a district-level mental health care plan. In this paper, we present an analysis of common issues faced by the differently resourced PRIME country districts in order to highlight the challenges and opportunities for integrating mental health into primary care across settings.

## Methods

The PRIME program has been described elsewhere [18] but relevant details are outlined below.

### Settings

The PRIME countries were selected intentionally to represent diverse settings that would give information about the range of approaches needed to implement mental health care. See [18] for further detail. The PRIME countries are from two continents (Africa and Asia), have different resource levels (Upper middle-income (South Africa), lower middle-income (India) and low-income (Ethiopia Uganda and Nepal)) and include a fragile state (Nepal). The countries share political commitment or intention to scale up of mental health care, a recognised prerequisite for service expansion [15], with Ministry of Health representatives from each country as project collaborators. The country districts are Sodo (Ethiopia), Kamuli (Uganda), Chitwan (Nepal), Sehore (India) and Dr Kenneth Kaunda district (Dr KK district) (South Africa). As far as possible, these districts were selected to be representative of the wider country context; none were existing sites for mental health research and none contained national academic centres for mental health. However, because of the fragility of the setting, the Chitwan district (Nepal) was selected on the basis of logistical feasibility and is considered to be relatively better-resourced than the rest of the country.

### Study design

A cross-sectional situation analysis largely relying upon information available in the public domain and supplemented by contact with key officials and service heads.

### Situation analysis tool

For the purposes of the PRIME project, we developed a new situation analysis tool (downloadable from http://www.prime.uct.ac.za/index.php/research/tools.html). Existing methods and tools for appraising mental health systems and services in LMICs did not meet the needs of PRIME either due to reliance on primary research [19], [20] or because they were not applicable to small population units, such as districts and sub-districts [21].

The situation analysis was orientated around those factors required for implementation of the WHO's mhGAP-IG [11] in the PHC setting [15]. A small number of items overlapped conceptually with items from the WHO-Assessment Instrument for Mental Health Systems (WHO-AIMS) [21]. Where duplication occurred, the WHO-AIMS item was included in preference. Methodology for in-depth case studies of mental health services in LMICs was also reviewed to identify further domains [19]. The draft situation analysis tool was circulated within the PRIME Research Programme Consortium, which includes expertise in health service evaluation, planning and implementation of mental health care services, and both multi-sectoral and community engagement in mental health care. To ensure a comprehensive approach feedback was also solicited from other experts in the field.

The situation analysis tool comprised six sections:

- Section I (*Relevant context; 39 items*) covered environmental, population, economic, health (including reproductive health and HIV) and social indicators.
- Section II (*Mental health politics, policies and plans; 15 items*): national and local political support for mental health care, including budgets, policies, plans, legislation and welfare benefits, and details of the specialist mental health workforce at national level.
- Section III (*Mental health treatment coverage; 19 items*): prevalence of MNS disorders, numbers receiving care and estimated treatment coverage.
- Section IV (*District level health services; 62 items*): general health services, including the available cadres of health professionals at each level in the system, specialist mental health services, with particular reference to elements of specialist mental health care needed to support PHC-based mental health care and delivery of the mhGAP packages [11], [22], the extent and nature of mental health care delivered in primary care and health systems structures to support integration of mental health care into PHC.
- Section V (*Community; 17 items*): sociocultural aspects, relevant non-health sector organisations and awareness-raising activities.
- Section VI (*Monitoring and evaluation; 4 items*): health information systems, and monitoring and evaluation of services.

For sections I-III, information was collected for both the national and district level to be cognisant of national programs even if these were absent in the district selected for the PRIME project. A section was also included for a narrative description of the district.

The situation analysis tools were completed by project co-ordinators and research staff from the PRIME countries between October and December 2011 and then reviewed by CH and MDS for completeness and comprehensibility. Discrepancies between data sources were noted, prompting cross-checking by project co-ordinators where possible. Concerns about the veracity of the data obtained from a particular data source were noted. Sources of information contributing to the situation analysis were documented, together with the date of data collection, thus allowing the document to be updated when new information became available.

### Ethics statement

Ethical approval was obtained from the Institutional Review Board of the College of Health Sciences, Addis Ababa, Ethiopia, but was not required from the other PRIME countries due to reliance on information available in the public domain. All data collected for the situation analysis were aggregated. Interviews were only conducted with people within their professional capacity as health administrators.

## Results

Information for completion of the situation analyses was drawn from a variety of sources in each country, including health facility and system records, health surveillance data, research publications, governmental and non-governmental reports, and supplemented with personal communication with key service co-ordinators. The data sources are listed by country in Table S1. Country teams reported their uncertainty about some sources of information, particularly data obtained as part of routine health service monitoring. The most incomplete section of the situation analysis was that relating to the community due to the lack of routinely collected data.

### The broader district contexts

There was a wide diversity of ethnicities, religions and languages in each of the districts. See table 1. In none of the districts could literacy be assumed, and was recorded as particularly low in Sodo (Ethiopia) (22%). Dr KK district (South Africa) was characterised by both high density urban populations and low density rural populations. A general lack of infrastructure was identified as a problem across sites, but particularly in Sodo (Ethiopia) where most of the population did not have electricity, sanitation or access to clean water within their homes.

**Table 1:** The context of mental health care scale-up across PRIME districts.

	Ethiopia	Uganda	India	Nepal	South Africa
District name	Sodo (Gurage Zone)	Kamuli	Sehore (Madhya Pradesh State)	Chitwan	Dr Kenneth Kaunda (North West Province)
District population	161,952	740,700	1,311,008	575,058	632,790
% rural	90%	97%	81%	73%	14%
Population density(persons/km^2^)	187	222	199	259	55
Ethnic diversity	>4 ethnic groups	>4 ethnic groups	1 ethnic groups	>9 ethnic groups	>4 ethnic groups
Linguistic diversity	>4 languages	3 languages	1 language	>8 languages	>4 languages
Religious diversity	Predominantly Christian	Christian and Muslim	Predominantly Hindu and Muslim, but also Christian and Sikh	>7 religions	Predominantly Christian
Literacy	22%	63%	71%	70%	88%
% households with electricity	Only available in urban setting	Unknown	91%	69%	82%
% households with functioning latrine	5%	70%	23%	80%	76%
% households with clean water supply	20%	57%	80%	86%	97%
Top five reasons for out-patient visits	Malaria	Malaria	Acute diarrhoea	Impetigo/boils	HIV
	URTI	URTI	Food poisoning	URTI	Hypertension
	Diarrhoea	Intestinal parasites	Measles	LRTI	Tuberculosis
	LRTI	STIs &HIV/AIDS	Chicken pox	Falls/injury	Asthma
	Intestinalparasites	Diarrhoea	Dengue	Otitis media	Diabetes
MNS disorders in top 10 out-patient visits	No	No	No	No	Yes (Epilepsy-6^th^, mental disorders-10^th^)
HIV prevalence	<1%	6.5%	<1%	<1%	30%
Other important contextual public health factors	Undernutrition, reproductive health	Increasing burden of NCDs	Infant and maternal mortality, family planning and communicable diseases	Dengue outbreaks, post-conflict health	High burden of infectious chronic diseases (HIV & tuberculosis) and concomitant rising burden of NCDs

MNS disorders = Mental, neurological and substance use disorders; URTI = Upper Respiratory Tract Infection; LRTI = Lower Respiratory Tract Infection; STIs = Sexually transmitted Diseases; HIV = Human Immunodeficiency Virus; AIDS = Acquired Immunodeficiency Syndrome; NCDs = Non-Communicable Disorders.

The health facilities in the districts were mostly concerned with providing services for acute communicable diseases, except in South Africa where chronic communicable diseases and non-communicable diseases (NCDs) predominated. Only in Dr KK district (South Africa) were MNS disorders among the top ten reasons for out-patient attendance. The burden of HIV was high in Dr KK district (South Africa) (30% adult seroprevalence) and Kamuli (Uganda) (6.5% adult seroprevalence) but was a much lower health priority in the other countries (<1% seroprevalence). Other notable public health issues burdening existing health services were outbreaks of dengue fever in Chitwan (Nepal), undernutrition and reproductive health issues in Sodo (Ethiopia) and emerging NCDs in Kamuli (Uganda).

### General and PHC context for integrating mental health care

The health system infrastructure varied across the PRIME districts, including the definition and scope of primary health care. See Table 2. In Sodo district (Ethiopia), only PHC services were present, staffed by non-physicians and mostly limited to out-patient care with a strong focus on prevention and promotion. In all other PRIME districts, secondary health care services were present, including hospitals staffed by medical doctors.

**Table 2:** General and primary health care context for integrating mental health care in PRIME districts.

	Ethiopia	Uganda	India	Nepal	S. Africa
Health facilities within the AHU					
Hospitals	0	2	2	2	4 (+ 1 mental hospital)
Community health centres	0	0	5	0	9
Primary care clinics	8	41	15	4	28
Sub-health centres	0	0	152	0	0
Health Posts	58	0	0	5	0
Sub-health posts	0	0	0	41	0
Other	None	None	None	None	15 mobile clinics
Available cadres of facility-based PHC workers for mental health care					
Doctors	No	Yes	Yes	No^1^	Yes
Non-physician clinical officers	Yes	Yes	Yes	Yes	No
Nurses	Yes	Yes	Yes	Yes	Yes
Psychologists/counsellors	No	No	Yes	No	Yes
Social workers	No	No	Yes	No	Yes
PHC worker training in mental health					
Pre-service	Limited, with minimal clinical exposure	Very limited (<1 week)	None	None	Yes (20% of nurse training)
In-service	None	None within last 2 years	One-off training for selected medical officers and frontline workers	Small number of PHC workers received five days training in last year	Limited
Psychotropic medications in PHC					
Antipsychotics	None	Chlorpromazine	None	None	All in WHO EDL^2^
		Haloperidol			
Antidepressants	None#	Imipramine	None	None	All in WHO EDL
		Amitriptyline			
Mood-stabilisers	None	None	None	None	All in WHO EDL
Anxiolytics	Diazepam	Diazepam	Diazepam	Alprazolam^1^	All in WHO EDL
Antiepileptics	Phenobarbitone	Phenytoin,	Phenytoin	Phenobarbitone^1^	All in WHO EDL
		Phenobarbitone			
Availability of psychotropic medications in PHC					
Reliable supply	No	No	No	No	Yes
Affordability	Only free for those exempted due to extreme poverty (<20%)	All available medications are free	All PHC services are free	All available medications are free	All PHC services are free

1 In Nepal, doctors and psychotropic medications are only available at the highest level of primary care, which is not locally accessible for the majority of the population and differs from the definition of PHC in the other country settings.

2 World Health Organisation's Essential Drug List.

Pre-service training of PHC workers in mental health care was limited to a few hours or days in all districts except Dr KK district (South Africa) where it formed a substantial percentage of the training time (20%). No in-service training had been conducted in Sodo (Ethiopia) or Chitwan (Nepal) districts. In Kamuli (Uganda), Sehore (India) and Dr KK district (South Africa), in-service training was sporadic and not comprehensive in terms of personnel.

The full range of psychotropic medication recommended by the WHO, and included within the essential drug lists of all PRIME countries, was only available in PHC services in Dr KK district (South Africa). In Sodo (Ethiopia) and Sehore (India) districts, psychotropic medication was limited to antiepileptic medication and benzodiazepines (anxiolytic medication). In the low-income country sites the supply of psychotropic medications was considered to be unreliable. All districts except Sodo (Ethiopia) provided free access to medication at the PHC level, including psychotropic medication when available. In Sodo (Ethiopia) medication costs were mostly borne by patients and their families on an out-of-pocket basis. Primary care-based, non-physician health workers who had received appropriate training were permitted to prescribe psychotropic medication in all districts except Sehore (India), but not permitted officially to diagnose or treat mental disorders independently except in Kamuli (Uganda) [23].

### Existing mental health care provision in PRIME districts

#### (i) Specialist mental health services

See Table 3. In Sodo (Ethiopia) there was no specialist mental health care provision within the district, with the nearest out-patient service 30 to 50 km away staffed by two psychiatric nurses serving a population of over one million people. In Kamuli (Uganda), one psychiatric clinical officer and one psychiatric nurse served a population of three-quarters of a million. In Chitwan (Nepal) two public-sector psychiatrists served the district population of over half a million, but also provided care for people from neighbouring districts. The central location of specialist care meant that it was inaccessible to many people due to the geographical terrain. In Sehore (India), within the public sector, one psychiatrist, one clinical psychologist, one generic counsellor and one mental health support worker served a population of 1.3 million. The Dr KK district (South Africa) was relatively better served by specialist mental health care, with a hospital specialising in care for neurological and psychiatric disorders, general hospitals with capacity for acute admissions for psychiatric crises and a multi-disciplinary team providing out-patient care, including outreach, for people with severe mental disorders. In Sehore (India) and Dr KK district (South Africa) multi-disciplinary mental health workers were also found in non-governmental organisations (NGOs) and the private sector.

**Table 3:** Baseline mental health care in PRIME districts.

	Ethiopia	Uganda	India	Nepal	S. Africa
Specialist mental health services in district					
In-patient mental health facilities	None	None	Acute admissions to the district general hospital.	Within general hospitals: 5 beds (public hospital in district capital), 25 beds (NGO), 48 beds (private)	1 public hospital providing specialist care for MNS disorders. Acute admission to 4 general hospitals
Out-patient mental health facilities	None	Yes	Yes	None (public)Yes (private, NGO	Yes
Psychological therapies	None	None	Yes, generic counselling	Private hospital – group therapy, motivational interviewing	Yes. A range of therapies offered according to professional preference and training. CBT is commonly used at specialist facility.
Alcohol detoxification	None	None	Not in public sector. One NGO de-addiction centre.	2 hospital-based facilities (1 public, 1 private)	1 public, 1 private facility (86 beds)
Mental health rehabilitation	None	None	None	None	Yes
Mental health workers in district					
Psychiatrists	0	0	1 (public)	2 (public), 3 (private) (in district capital)	2 full time psychiatrists in psychiatric hospital who also provide district outreach services part-time
Neurologists	0	0	0	0	1
Psychiatric clinical officer/practitioner	0	1	1 (private)	N/A	0
Psychiatric nurses	0	1	0	0 (public), 4 (private)	No dedicated psychiatric nurses
Clinical psychologists	0	0	1 (public), 2 (NGOs)	0	1 at PHC, 3 in district hospitals. 5 in specialist facility which also has 3 Psychology interns
Counsellors	0	0	1 (public), 1 (NGO)	0 (public), 7 (private)	139 (lay health worker counsellors for pre-post HIV testing, behaviour change and adherence counselling)
Mental health social workers	0	0	1 (NGO)	0	2 or 3
Mental health occupational therapists	0	0	1 (NGO)	0	0
Mental health support workers	0	0	1 (public), 1 (NGO)	0	0
Existing mental health care in PHC					
Current actions by prescribers in PHC	SMD identification and referral. Epilepsy follow-up care following specialist review.	Identification and referral of SMD	No intervention for SMD or Depression. Epilepsy treatment initiated.	SMD identification and referral. Prescription of benzodiazepines for depression. Epilepsy treatment initiated.	SMD identification, prescription of psychotropic medication and referral.
Current actions by non-prescribers in PHC	None	Identification and referral	None	Identification and referral	Identification and referral
Availability of evidence-based psychosocial interventions in PHC					
Problem-solving, behavioural activation	None	None	None	None	Limited service by the one available psychologist
Interpersonal psychotherapy, cognitive behavioural therapy, motivational interviewing	None	None	None	None	None

PHC = Primary health care; MNS disorders = Mental, neurological and substance use disorders; NGO = non-governmental organisation; SMD = severe mental disorders; HIV = human immunodeficiency virus.

In-patient psychiatric care was unavailable in Sodo (Ethiopia) and Kamuli (Uganda) districts. Public sector services for people with alcohol disorders were only available in Chitwan (Nepal) and Dr KK district (South Africa).

Public sector psychological therapies were absent in the low-income country districts (in Ethiopia, Nepal and Uganda) and were limited to a small number of clinical psychologists and general counsellors in the middle-income country districts (in India and South Africa), although provision from NGOs and the private sector was better in these settings. Facility-based mental health rehabilitation services were only available in Dr KK district (South Africa).

#### (ii) Mental health care in PHC at baseline

There was evidence of very limited delivery of mental health care in PHC settings across the country sites. In the Dr KK district (South Africa) PHC workers provided follow-up clinical care for people with chronic and severe mental health problems, including continuing the prescription of medication, psycho-education and some counselling. In the other country sites, when present, PHC activities were largely limited to identification of people with severe mental disorders and direct referral to specialist mental health services without any treatment intervention. For epilepsy, there was evidence of more active intervention within PHC, with initiation of prescribing of antiepileptic medication in Sehore (India) and Chitwan (Nepal) and continuation of antiepileptic medication initiated by specialists in Sodo (Ethiopia). Interventions for depression and other common mental disorders in PHC were extremely limited across sites. Evidence-based psychological therapies (World Health Organisation, 2010) were not available in any of the PHC facilities in the PRIME districts except for DR KK district (South Africa) where problem solving and behaviour activation therapy was available from one psychologist.

#### (iii) Treatment coverage for MNS disorders

Information on treatment coverage for the PRIME priority MNS disorders was difficult to access and not generally considered to be reliable. Epidemiological estimates of community prevalence of the priority MNS disorders were available for the district neighbouring Sodo in Ethiopia [24]–[27]. In Chitwan (Nepal), community prevalence estimates were available for depression and other common mental disorders [28], and similarly for the North West province in which Dr KK district (South Africa) is located [29]. For the remaining MNS disorders and in the other PRIME sites, it was necessary to rely on extrapolation from national prevalence estimates (India, South Africa) or studies conducted in other LMICs (Uganda, Nepal). Estimates of numbers of people with MNS disorders in the district accessing care were not available in Kamuli (Uganda) and Sehore (India) and only limited information was available in Sodo (Ethiopia), Chitwan (Nepal) and Dr KK district (South Africa). Although all districts had theoretical mechanisms for recording treatment episodes for persons with mental disorders (see Table 4), collated figures for each district were either not available or deemed incomplete or too unreliable to allow confident estimates of treatment gap. On the basis of the available data, it was apparent that treatment coverage was extremely low, except in the South African district where treatment coverage in the form of psychotropic medication for severe mental disorders was thought to be moderate.

**Table 4:** Health service organisation to support mental health care in PRIME districts.

	Ethiopia	Uganda	India	Nepal	South Africa
Mental health service organisational structures within the district					
District mental health plan or implementation of national mental health plan	No	No	No, but there is a mental health programme	No	Yes
Implementation of mental health legislation	No	No	Yes	No	Yes
Budget for mental health (% of district health budget)	No	No	No	No	Yes (not ring-fenced)
Mental health co-ordinator	No	No	No	Yes	Yes
Mental health service organisation in PHC					
Mental health part of PHC basic packages	Yes, but not implemented	Yes, but limited implementation	Yes, but not implemented	No	Yes, implemented in practice
Information system for recording MNS disorders	2 categories: ‘mental or behavioural disorder’ and ‘epilepsy’	7 mental health conditions included in HMIS	Not in HMIS. Categories of ‘mild, moderate and severe’ disorders.	7 mental health conditions included in HMIS	No specific disorders recorded.Mental health visits (<18/≥18 years), Mental health admissions (<18/≥18 years),Mental health involuntary admission rate
Monitoring and evaluation systems for quality of mental health care	No	No	No	No	Yes
Screening tools for MNS disorders	No	No	No	No	Yes
Register of persons with MNS disorders	No	No	No	Yes	Yes
Intervention guidelines for MNS disorders	No	No	No	No	Yes
Mechanism for detecting drop-out from mental health care	No	No	No	No	Yes, through CHW tracing
Training manuals for mental health	No	No	No	No	Yes
Staff supervision structures for mental health	No	No	No	No	Yes
Interface between PHC and specialist mental health services					
Referral from PHC to specialists	Yes	Yes	Yes	Yes	Yes
Back-referral from specialists to PHC	No	No	No	No	Yes
Phone consultation	No	No	No	No	No
Face-to-face meetings	No	No	No	No	Yes
Models of care for chronic disorders in PHC					
Adherence support	Yes, for HIV & TB	Yes, for HIV & TB	Yes, for HIV & TB	No	Yes, for HIV
Outreach for loss to follow-up	Yes, for HIV	Yes, for HIV	Yes, for HIV	No	Yes, for HIV, TB and SMD
Non-health sector supports for mental health care					
Disability payments	No	No	Yes, for SMD	Yes, for SMD	Yes, for SMD and epilepsy

PHC = primary health care; Health Management Information System; MNS disorders = mental, neurological and substance use disorders; Community Health Workers; HIV = Human immunodeficiency virus; TB = tuberculosis; SMD = severe mental disorders.

### District-level health service organisation for mental health care in PHC

See Table 4. Despite the presence of national mental health policies or programmes in South Africa, India and Nepal, only one sub-district of Dr KK district (South Africa) and Sehore (India) had initiated the implementation of plans, legislation and making funds available at the district level. All countries had included mental health among the basic health services delivered in PHC, but this was reported to be implemented only in Dr KK district (South Africa). Health Management Information System (HMIS) indicators specific to mental disorders were present in some countries: Kamuli (Uganda) and Chitwan (Nepal) districts both had seven categories of MNS disorders included within the HMIS. However, In Sodo (Ethiopia), Sehore (India) and Dr KK district (South Africa), very limited categories were available for reporting. A dedicated mental health co-ordinator was absent in all districts except Dr KK district (South Africa) and Chitwan (Nepal).

Most of the service organisation components considered important to support delivery of mental health care in PHC were absent from most districts. Specifically, there were no PHC worker training manuals, screening tools or guidelines for interventions for MNS disorders, mechanisms to register people with severe mental disorders and detect drop-out from care, supervision systems or monitoring and evaluation systems for mental health care in Sodo (Ethiopia), Kamuli (Uganda), Sehore (India) or Chitwan (Nepal).

Nonetheless, all districts had relevant models of PHC for chronic communicable disorders, specifically tuberculosis (TB) in all districts and HIV in most districts. Service characteristics for these conditions included adherence support and outreach for patients lost to follow-up. Disability payments were, in principle, available for people with severe mental disorders in Sehore (India), Chitwan (Nepal) and Dr KK district (South Africa) but absent from the Ethiopian and Ugandan sites, although low levels of awareness of entitlement in Chitwan (Nepal) meant that few eligible people benefitted in practice.

The interface between mental health specialists and PHC was mostly limited to paper referrals from PHC to specialist care, except in Dr KK district (South Africa) where back-referrals from specialist care to PHC and face-to-face meetings with mental health specialists, including provision of supervision, were reported.

### Community engagement and care

See Table 5. Community-level awareness about MNS disorders and the existence of effective treatments (sometimes termed “mental health literacy” [30]) was recognised to be limited across the PRIME districts, with strong reliance on self-help, informal healing approaches, and more formal traditional and religious healing. In Ethiopia, information about sociocultural explanatory models and help-seeking behaviours in the neighbouring district was available from published research studies [31]; however, detailed information about the extent of help-seeking from traditional and religious healers for the different MNS disorders was mostly lacking across sites, particularly for alcohol use disorders. Models of collaboration between PHC services and traditional and religious healers were absent from all PRIME districts.

**Table 5:** Community context for scaling up mental health care in districts in PRIME study countries.

	Ethiopia	Uganda	India	Nepal	South Africa
Interface between PHC and community					
Community-based PHC workers (paid)	Yes (1 per 2500 population)	No	Yes: Accredited social health activists (1 per 1000), DOTS providers	No	Yes (n = 1577), Includes DOTS providers, adherence supporters, health educators
Community-based health volunteers	Yes	Yes, identify and refer.	Yes, for HIV care: outreach workers for people dropping out of care and peer educators	Yes (1 per 1000 population)	No
Links between PHC and traditional or religious healers	None	None	None	None	None
Formal support for families	None	Minimal	None	None	Yes
Use of traditional/religious healers					
Psychosis	High [31]	High	High	High	High
Depression	Low [31]	Low	High	Low	Unknown
Alcohol use disorders	Low	Low	High	Low	Unknown
Epilepsy	High [31]	High	High	High	Unknown
Community attitudes					
Stigma	High [32], [33]	High	High	High	Significant
Abuse	Chaining in homesStones thrown [32], [33]	Common, in many forms	Restraint at home	Chaining in homesStones thrown	Financial exploitation
Community resources					
Peer support/self-help groups	None	Yes, livelihoods for persons with mental disorders	Yes, peer educators for HIV	None	Alcoholics anonymous
NGOs, FBOs or CBOs working with persons with MNS disorders	None	None	Only substance use	Only substance use	Yes, providing limited social support and carrying out advocacy work for persons with severe mental disorders and intellectual disabilities.
Supported housing	None	None	None	None	No
Community-based rehabilitation	None	None	None	None	Very limited
OTHER					Court-linked counselling for alcohol problems

DOTS = Directly Observed Treatment – Short-course; NGO = Non-governmental organization, FBO = faith-based organisation, CBO = Community-based organization.

Stigma towards, and abuse of, persons with MNS disorders was noted in all PRIME districts, with research evidence from Ethiopia [32], [33]. The nature of abusive practices varied across sites, with use of physical restraints in Sodo (Ethiopia), Kamuli (Uganda), Chitwan (Nepal) and Sehore (India), while financial exploitation was thought to be the more prominent form of abuse in Dr KK district (South Africa). In all sites, the problem of homeless persons with MNS disorders was apparent to investigators but not quantified.

In Sehore (India), Sodo (Ethiopia) and Dr KK district (South Africa), paid community health workers formed a reasonably robust interface between facility-based PHC services and the community. Volunteer health workers were also present in most of the districts. In none of the sites were these community health workers engaged in mental health care.

NGOs, faith-based organisations and community-based organisations were present in all districts, but were only working with persons with mental disorders in Kamuli (Uganda) and Dr KK district (South Africa), and with persons with substance use disorders in Chitwan (Nepal) and Sehore (India). Self-help groups relating to MNS disorders were only present in Dr KK district (South Africa) for alcohol use disorders and in Kamuli (Uganda) for people with all types of mental disorders. Only in the Kamuli (Uganda) district was a community organisation working to improve the livelihoods of persons with MNS disorders [34]. Public sector sheltered housing was absent in all districts. Apart from very limited provision within Dr KK district (South Africa), there were no community-based rehabilitation services for persons with MNS disorders in the PRIME districts.

## Discussion

The PRIME situation analysis tool provided systematic information on the context (national and district), the existing structures and systems to support integration of mental health care into PHC, and the challenges and potential opportunities in the five country sites. The PRIME study districts face substantial contextual and health system challenges in developing and implementing mental health care plans that will be able to deliver adequate services for people with priority MNS disorders in primary care. Although many difficulties were more pronounced in the low-income country settings, common challenges and opportunities were seen across the range of PRIME countries.

A previous situation analysis [20] of three sub-Saharan African countries (Ghana, South Africa and Uganda) examined the extent of implementation of recommendations for integrating mental health into PHC outlined in the 2001 World Health Report [35]. In our study, by going beyond mental health service components and also examining the broader community and health service context, we were additionally able to identify likely system constraints and opportunities for integrating mental health care. Furthermore, the PRIME situation analysis tool had the advantage of relying largely on publically available data with no need for primary research data.

A limitation of the PRIME study approach was that the situation analysis may not have given a fully accurate picture of the situation on the ground; for example, potentially under-estimating the numbers of people with MNS disorders already receiving care in PHC due to poor information systems. We attempted to overcome this limitation by triangulating sources of data and contacting key service co-ordinators and planners to provide clarification where possible. An additional limitation was that the PRIME situation analyses necessarily focused on readily measurable health system structures and, in the absence of previously published reports, were unable to give an in-depth understanding of attitudinal factors, for example, acceptability to PHC workers of task sharing mental health care. This will be addressed in other qualitative formative research, being conducted by the PRIME study [18]. Nonetheless, in the context of limited time and resources to undertake primary research, the PRIME situation analysis tool yields data that has value for the planning of integrated mental health services, as well as evaluating the impact of implementing these plans.

### Cross-country challenges

Across all PRIME country districts, competing public health priorities, financial constraints and high levels of poverty and social deprivation are enormous challenges to the delivery of mental health care and define the context in which policy-makers and planners need to be engaged. Limited levels of community awareness and high levels of stigma and abuse also emerged as cross-country challenges requiring intervention in order to improve demand for services, retention in services and support for people with MNS disorders. Health system structures and systems to support mental health care were also largely absent; dedicated service co-ordinators and budget, reliable supplies of medications, decision-support tools for clinicians, and monitoring and evaluation mechanisms will all need to be introduced as part of a comprehensive and workable mental health care plan.

Three other challenges seen across country sites will now be considered in greater depth: deficient information systems, lack of specialist mental health professionals and the feasibility of psychosocial care.

### Deficient information systems

Comprehensive information about treatment coverage for MNS disorders was lacking across all sites. Being able to track changes in treatment coverage for priority MNS disorders is essential for monitoring the impact of initiatives to implement and scale-up mental health care. Health system interventions to improve the recording and reporting of service utilisation are, therefore, needed alongside interventions to train PHC workers to deliver mental health care. Further research is needed to identify optimal service utilisation indicators for mental health care integrated into PHC in LMICs. The situation analysis also revealed the absence of information on the equitable distribution of treatment coverage; for example, for women, the poor and those living in rural areas. Poverty is an established determinant of MNS disorders, as well as a consequence of mental ill-health, meaning that persons with MNS disorders are often to be found amongst the poorest in society [6]. Given the restrictions on access to free PHC services in Sodo (Ethiopia) and absence of disability payments for those with severe mental disorders in both Sodo (Ethiopia) and Kamuli (Uganda) districts, the extent to which poverty is an obstacle to accessing care in these districts needs to be evaluated. Beyond the impact of gender, monitoring the equitable distribution of treatment coverage is likely to require primary research to supplement that available from routine service indicators.

### Lack of specialist mental health care

The scarcity of specialist mental health workers to support the delivery of mental health care by PHC workers was evident across all sites, except in some sub-districts of the Dr KK district (South Africa) that were relatively well resourced but still unable to meet the need. The acute shortage of mental health professionals across LMICs is well documented [36] but starkly revealed in the PRIME settings which were intentionally selected to represent the conditions faced by the majority of people in each country, rather than locating the research sites close to specialist facilities or research centres. Studies have shown repeatedly that, without some form of supportive supervision by specialist mental health professionals, it is not possible to achieve sustainable integration of mental health care into PHC of an adequate quality [14], [15], [37]. As well as increasing the absolute numbers of specialist mental health professionals there also needs to be political support for a shift in their role from direct delivery of services to a focus on training and supervision [15]. In order to achieve adequate levels of supervision and support from specialist services in the PRIME sites, lobbying for additional specialist mental health workers or using innovative methods, such as peer support or remote supervision using telemedicine, will need to be considered.

### Feasibility of psychosocial care

Going beyond the absolute numbers of mental health professionals, there is also the challenge of non-availability of a multi-disciplinary workforce trained to provide psychosocial care. Public sector psychologists, social workers, counsellors and support workers were in short supply. The evidence-based psychosocial interventions advocated within the mhGAP intervention guide may not be deliverable on the ground if appropriately skilled health personnel are not available to deliver and supervise such care. PHC nurses and community-based health workers have been shown to be able to provide effective circumscribed psychosocial interventions in LMIC settings [38], [39], as well as psychosocial interventions implemented outside the health system as evidenced in Nepal [40], but it remains to be seen whether this is feasible outside of a research project. Again, more innovative approaches may be required. There is preliminary evidence from Uganda that traditional healers can be trained to deliver basic psychosocial interventions [34], but the lack of collaborative frameworks between health systems and traditional and religious healers may limit the feasibility of this approach in the PRIME settings. In Ethiopia, there are successful, NGO-led community-based rehabilitation programmes within which mental health care could be integrated [41], although they are not currently operating in the PRIME site, and setting up parallel systems to the existing health service is unsustainable. Expansion of the evidence base on psychosocial interventions that can be delivered the setting of a busy PHC clinic is sorely needed and will be a focus of PRIME in Ethiopia, Nepal and South Africa.

### Cross-country opportunities

A clear opportunity identified within each of the PRIME districts was the presence of existing services for chronic disorders. Depression and alcohol use disorders, in particular, have been shown to be highly prevalent in people with chronic infectious and non-communicable disorders [42]. Improving detection and management of mental disorders through the integration of mental health care into platforms of care delivery for HIV, TB and other NCDs has the potential to improve both physical and mental health outcomes [43], [44]. For psychotic disorders and epilepsy, specific models of chronic care are likely to be required [37], but can make use of the structures and systems of existing chronic disease services in order to maximise retention in care and enhance medication adherence. Models of chronic care, for example, the Innovative Care for Chronic Conditions Framework [45], offer the potential to be more patient-centred, comprehensive, collaborative and proactive than care models for acute illnesses. Furthermore, certain aspects of care for chronic disorders, such as self-help groups, service user and family participation and attention to PHC worker burnout may be relatively better developed in the field of mental health care. With the integration of mental health into PHC the care of all chronic disorders could be enhanced.

### Conclusions

The low level of baseline health system preparedness across sites underlines that interventions at the levels of health care organisation, health facility and community will all be essential for sustainable delivery of quality mental health care integrated into primary care. Such a multi-faceted approach will be evaluated in the implementation of the PRIME mental health plans.

## Supporting Information

Table S1 **Data sources for the PRIME situation analysis.**
(DOC)
