# Supplementary material for: Challenges and Opportunities for Implementing Integrated Mental Health Care: A District Level Situation Analysis from Five Low- and Middle-Income Countries
Source: PLoS One. 2014 Feb 18;9(2):e88437. doi: 10.1371/journal.pone.0088437 (PMC3928234; doi:10.1371/journal.pone.0088437)
Supplement: Table S1 — Data sources for the PRIME situation analysis. (DOC) [file pone.0088437.s001.doc]

**Table S1: Data sources for PRIME situation analysis**

| **Ethiopia** |
| --- |
| **Grey literature**  Central Statistical Agency [Ethiopia] and ICF International (2012) Ethiopia Demographic and Health Survey 2011. Addis Ababa, Ethiopia and Calverton, Maryland, USA: Central Statistical Agency and ICF International.  Central Statistical Authority (CSA) (2008) Summary and statistical report of the 2007 population and housing census. Population size by age and sex. Addis Ababa: Population Census Commission, Federal Democratic Republic of Ethiopia.  Federal Democratic Republic of Ethiopia Ministry of Health (2012) National Mental Health Strategy, 2012/13-2015/16. Addis Ababa: Ministry of Health.  mhGAP-Ethiopia Working Group (2010) Mental Health Gap Action Programme in Ethiopia: final document. Addis Ababa: Ministry of Health, Ethiopia.  World Health Organization (2005) World Health Organization Assessment Instrument for Mental Health Systems. Geneva: WHO.  **Websites**  <http://www.indexmundi.com/ethiopia/literacy.html> (accessed November 2012)  <http://water.worldbank.org/water/> (accessed November 2012)  <http://www.geni.org/globalenergy/library/energy-issues/ethiopia/index.shtml> (accessed November 2012)  <http://www.who.int/countries/eth/en/> (accessed November 2012)  <http://www.unicef.org/infobycountry/ethiopia_statistics.html> (accessed November 2012)  **Routinely collected health indicators**  Sodo District Health Office report, 2010/2011  **Research publications**  Alem A, Jacobsson L, Araya M, Kebede D, Kullgren G (1999) How are mental disorders seen and where is help sought in a rural Ethiopian community? A key informant study in Butajira, Ethiopia. Acta Psychiatrica Scandinavica Supplement 397:40-47.  Awas M, Kebede D, Alem A (1999) Major mental disorders in Butajira, southern Ethiopia. Acta Psychiatrica Scandinavica, Supplement 99(397):56-64.  Fekadu A, Alem A, Hanlon C (2007) Alcohol and drug abuse in Ethiopia: past, present and future. African Journal of Drug and Alcohol Studies 6(1):39-53.  Haile, E (2010) Knowledge, attitudes and perspectives of primary health care workers regarding integration of mental health into primary health care: a study of selected health centres in Addis Ababa, Ethiopia. Unpublished study for completion of psychiatry qualifying examination.  Kebede D, Alem A, Shibre T, et al. (2003) Onset and clinical course of schizophrenia in Butajira-Ethiopia. A community-based study. Social Psychiatry and Psychiatric Epidemiology 38:625-631.  Negash A, Alem A, Kebede D, Deyessa N, Shibre T, Kullgren G (2005) Prevalence and clinical characteristics of bipolar I disorder in Butajira, Ethiopia: a community-based study. Journal of Affective Disorders 87(2-3):193-201.  Shibre T, Negash A, Kullgren G, et al. (2001) Perception of stigma among family members of individuals with schizophrenia and major affective disorders in rural Ethiopia. Social Psychiatry and Psychiatric Epidemiology 36(6):299-303.  Shibre T, Kebede D, Alem A, et al. (2003) Schizophrenia: illness impact on family members in a traditional society - rural Ethiopia. Social Psychiatry and Psychiatric Epidemiology 38:27-34.  Tekle-Haimanot R, Forsgren L, Ekstedt J (1997) Incidence of epilepsy in rural central Ethiopia. Epilepsia 38:541-546.  **Personal communications**  Federal Ministry of Health  Sodo District Health Office |
| **India** |
| **Grey literature**  Department of Public Health and Family Welfare, Madhya Pradesh, 11^th^ Five year Plan  Department of Public Health and Family Welfare (2006) Medium term Health Sector Strategy, Madhya Pradesh, India  Directorate General of Health Services, Ministry of Health and Family Welfare (2004) India, Mental Health an Indian Perspective 1946–2003  Government of India, Ministry of Health and Family Welfare, Maternal Health Guidelines  Government of Madhya Pradesh, Economic Survey Report, 2010-11  Government of India, Census report, 2011  Government of India, Department of Health and Family Welfare, State Health Policy (draft).  Government of India, Mental Health Act, 1987  Government of India, National Institute of Health and Family Welfare, National Mental Health Program  Government of India, The Mental Health Act-1987 (Revised draft)  Government of Madhya Pradesh, Essential drug list, 2011  Government of Madhya Pradesh, Madhya Pradesh, State Human Development Report, 2007  National AIDS Control Organization (2008) Operational Guideline for District AIDS Prevention Units  National Rural health Mission, Programme Implementation Plan, 2006 – 2012, Madhya Pradesh, India  WHO –AIMS REPORT on Mental Health systems in Uttarakhand, India, 2006  **Websites**  [http://mohfw.nic.in/NRHM/State_Profile.htm#mp](http://mohfw.nic.in/NRHM/State_Profile.htm#mp), (accessed November 2012) [http://mohfw.nic.in/NRHM/Health_Profile.htm#mp](http://mohfw.nic.in/NRHM/Health_Profile.htm#mp) (accessed November 2012)  <http://mohfw.nic.in/NRHM/Organogram/MP.htm> (accessed November 2012) <http://sehore.nic.in/trade_and_industries.htm> (accessed November 2012)  <http://mohfw.nic.in/NRHM/RKS.htm> (accessed November 2012)  [http://www.mohfw.nic.in/NRHM%20state%20and%20district%20health%20mission-institutional%20setup.htm](http://www.mohfw.nic.in/NRHM state and district health mission-institutional setup.htm), (accessed November 2012)  [http://mohfw.nic.in/NRHM/State%20Files/mp.htm](http://mohfw.nic.in/NRHM/State Files/mp.htm) (accessed November 2012)  <http://www.sehore.nic.in/trade_and_industries.htm> (accessed November 2012)    **Routinely collected health indicators**  Government of India, Special bulletin on maternal mortality, in India, 2007-09 SRS  Government of Madhya Pradesh, Basic Health Information & Indicators  MOHFW, National Family Health survey, GOI, District household and facility survey report 2007-08  NACO, Sentinel surveillance report, 2006  UNDP, Human Development reports of India, 2002 to 2007  UNDP, HDR, District Fact Sheet, 2007  MOHFW, NACO, M&E & research, surveillance, 2006  **Research publications**  Trivedi JK (2009) Mental Health Act, salient features, objectives, critique and future directions. Indian Journal of Psychiatry 11-19 |
| **Nepal** |
| Central Bureau of Statistic (CBS) (2003). Population Monograph of Nepal, Central Bureau of Statistics, government of Nepal, Kathmandu.  Department of Health Services (DoHS) (2010). Annual Report 2066/67 (2009/2010), Ministry of Health and Population, Department of Health Services, Kathmandu, Nepal  Department of Health Services (DoHS) (2063) Manual for Data Management Training 2063, Department of Health Services, Government of Nepal, Kathmandu.  District Development Committee (DDC) (2061). District Profile of Chitwan 2061, District Development Committee, Chitwan Nepal.  District Development Committee; District AIDS Coordination Committee (2063). District HIV/AIDS Plan 2063-2065, District Development Committee and District AIDS Coordination Committee, Chitwan, Nepal  District Public Health Office (DPHO) (2011). Annual Report 2010/11,District Public Health Office, Chitwan  DoWSS, (2067/68). Annual Report (2067/2068), Department of drinking water and Sanitation, Chitwan  Government of Nepal/WHO (2006) Mental health treatment and protection act, 2006/2063. Government of Nepal/World Health Organization, Kathmandu Nepal    Informal Sector Research and Study Center (ISRSC) (2008). District Profile of Nepal 2007/2008, Informal Sector Research and Study center, Kathmandu  Ministry of Health and Population ( MOHP), National Mental Health Policy, 1996, Kathmandu Nepal  Ministry of Health and Population (MOHP) (2009/2010) Fact Sheet: HIV epidemic update of Nepal, as of August 2010, National Centre for AIDS and STD Control, Ministry of Health and Population, Kathmandu  Ministry of Health and Population (MoHP) [Nepal], New ERA, and ICF International Inc. (2012). Nepal Demographic and Health Survey 2011. Kathmandu, Nepal: Ministry of Health and Population, New ERA, and ICF International, Calverton, Maryland.  Ministry of Health and Population (MOHP) [Nepal], New ERA, and Macro International Inc. (2007). Nepal Demographic and Health Survey 2006. Kathmandu, Nepal: Ministry of Health and Population, New ERA, and Macro International Inc.  Ministry of Health and Population (MOHP)[Nepal], Nepal Health Sector Program- Implementation Plan II (NHSP-IP2) ( 2010-2015)  Ministry of Health and Population(MOHP)[Nepal], National Health Policy, 2048 (1991)  Shakaya Dhana Ratna (2008), Suicide and Mental Illness Our Responsibility.  WHO and Ministry of Health and Population (MOHP) (2006),WHO-AIMS Report on Mental Health System in Nepal, WHO and Ministry of Health, Kathmandu, Nepal, 2006  **Routinely collected health indicators**  We visited many hospitals (both government. and private) in Chitwan and reviewed records/registration to explore the number of people visited in that hospital to seek MH services.  **Research publications**  Kohrt, BA, Speckman, RA, Kunz, RD, Baldwin, JL, Upadhaya, N, Acharya, R, Sharma, VD, Nepal, M K, Worthman, CM (2009) Culture in psychiatric epidemiology: using ethnography and multiple mediator models to assess the relationship of caste with depression and anxiety in Nepal. Annals of Human Biology 36(3), 261-280.  Luitel NP, Jordans MJ, Sapkota RP, Tol WA, Kohrt BA, Thapa SB, Komproe IH, Sharma B (TPO Nepal) (2013), Conflict and mental health: a cross sectional epidemiological study in Nepal, Kathmandu, Social Psychiatry and Psychiatric Epidemiology 48(2): 183-93  **Personal communication**  Psychiatrists, Primary Healthcare staff, District Public Health Office staff, Female Community Health Volunteers, Non-government Organisation staff and psychologists. |
| **South Africa** |
| 2008/2009 Annual Report Dr Kenneth Kuanda District  Census 2001 (2003) Investigation into appropriate definitions of urban and rural areas for South Africa: Discussion document/ Statistics South Africa. Pretoria: Statistics South Africa, 195p.Available at: www.statssa.gov.za/publications/P0302/P03022011.pdf  Census 2011 (2012) Statistical release –P0301.4. Statistics South Africa. Available at http://www.statssa.gov.za/census01/html/UrbanRural.pdf  Mid-year population estimates (2007) Statistical release –P032. Statistics South. Available at: www.statssa.gov.za/publications/P0302/P03022007.pdf  Mid-year population estimates (2011) Statistical release –P032. Statistics South Africa. Available at: http://www.statssa.gov.za/Publications/P03014/P030142011.pdf  North West Province of South Africa (2002) Human Health and Well-Being. In: State of the Environment Report. Available at: http://www.nwpg.gov.za/soer/FullReport/human%20health.html  Republic of South Africa and UNDP (no date) Report on Millennium Development Goal 5. Available at: http://www.statssa.gov.za/nss/Goal_Reports/GOAL%205-IMPROVE%20MATERNAL%20HEALTH.pdf  Lau (2009) Intimate Partner Violence. UNISA and MRC Fact Sheet. Available at:  www.mrc.ac.za/crime/intimatepartner.pdf  South African Department of Health (1997) White Paper for the transformation of the Health System in South Africa. Available at: http://www.doh.gov.za/show.php?id=3189#Chapter 12  WHO and Department of Psychiatry and Mental Health, University of Cape Town, South Africa (2007) WHO-AIMS Report on mental health system in South Africa.  South African Department of Health (2000) PHC Package for SA-Norms and standards. Available at: www.doh.gov.za/docs/policy/norms/full-norms.html  South African Government services (2004) Social Assistance Act 13, 2004. Available at: www.info.gov.za/view/DownloadFileAction?id=67950  **Websites**  <http://www.localgovernment.co.za/districts/view/40> (accessed November 2012)  <http://www.bbc.co.uk/news/world-africa-14094760> (accessed November 2012)  [www.stats.gov.cn/english/.../brics2011/](http://www.stats.gov.cn/english/.../brics2011/) (accessed November 2012)  [www.info.gov.za/view/DownloadFileAction?id=67950](http://www.info.gov.za/view/DownloadFileAction?id=67950) (accessed November 2012)  <http://www.statssa.gov.za/keyindicators/keyindicators.asp> (accessed November 2012)  **Research publications**  Norman R, Bradshaw D, Schneider M, Pieterse D, Groenewald P. Revised Burden of Disease Estimates for the Comparative Risk Factor Assessment, South Africa 2000. Methodological Note. Cape Town: South African Medical Research Council, 2006.  Mbokata M and Moodley J (2003). Domestic abuse - an antenatal survey at King Edward VIII hospital, Durban. South African Medical Journal 96:455-457  Lund C, Kleintjies S, Kakuma R, Flisher AJ (2010). Public sector mental health systems in South Africa: inter provincial comparisons and policy implications. Journal of Social Psychiatric Epidemiology 45:393-404.  Moutrie A and Kleintjes SR (2006) Women’s mental health in South Africa. In: Ijumba P, Padarath A (eds). South African Health Review 2006. Durban: Health Systems Trust.  Burn JK (2011). The Mental Health Gap in South Africa- A human rights issue.  The Equal Rights Review 6: 99-113.  Herman AA, Stein DJ Seedat S, Heeringa SG, Moomal H, Williams DR (2009). The South African stress and health study (SASH):12 month and lifetime prevalence of mental disorders. South African Medical Journal 99:339-344  Petersen I, Ssebunnya J, Bhana A, Baillie K, the MHaPP Research Programme Consortium (2011). Lessons from case studies of integrating mental health into primary healthcare in Uganda and South Africa. International Journal of Mental Health Systems, 5 (8). doi:10.1186/1752-4458-5-8  Campbell-Hall V, Petersen I, Bhana A, Mjadu S, Hosegood V, Flisher AJ (2010). Collaboration between Traditional practitioners and Primary Health Care staff in South Africa: Developing a workable partnership for Community Mental Health Services. Transcultural Psychiatry 47:610-628.  Kakuma R, Kleintjes S, Lund C, Drew N, Green A, Flisher AJ, d the MHaPP Research Programme Consortium (2010). Mental health stigma: what is being done to raise awareness and reduce stigma in South Africa? African Journal of Psychiatry 13: 116-124.  **Personal communication**  Information officer- STATSSA  Mental Health Co-ordinator:Tlokwe  Mental Health Co-ordinator: Matlosana  Provincial Mental Health Co-ordinator  District Information Officer, 2011 |
| **Uganda** |
| **Websites**  Kamuli District web portal. Accessible from <http://kamulidistrict.go.ug/>  **Grey literature**  Annual Health Sector Performance Report 2010/11  Annual Health Sector Performance Report, Health Sector Strategic Plan III, Kamuli District Development Plan (2007/8 – 2009/10)  Annual Health Sector Performance Report, Kamuli District Development Plan (2007/8 – 2009/10)  Health Management Information System (HMIS) returns  Health Sector Strategic Plan II, Kamuli District Council Score card 2008/09  HIV/AIDS Indicators Survey Database, Uganda PMTCT statistics, 2010  Kamuli Town Council Profile  National Health Policy, 2010  National Policy on Mental health, Neurological and substance abuse services, 2011  Police Annual Crime report 2010  STAR-EC report 2009  Uganda Bureau of Statistics report, Kamuli District Water Office  Uganda National Household Survey, Kamuli OVC Strategic Plan  United Nations Population Fund (UNFPA), 2011; Kamuli District Council Score card 2009; 2011  United Nations Population Fund (UNFPA), Kamuli District Council Score card 2008/09  **Routinely collected health indicators**  Quarterly HMIS returns  **Personal communication**  Nil |
